# Ice Control during Cryopreservation of Heart Valves and Maintenance of Post-Warming Cell Viability

**DOI:** 10.3390/cells11121856

**Published:** 2022-06-07

**Authors:** Kelvin G. M. Brockbank, John C. Bischof, Zhenzhen Chen, Elizabeth D. Greene, Zhe Gao, Lia H. Campbell

**Affiliations:** 1 Tissue Testing Technologies LLC, 2231 Technical Parkway, Suite A, North Charleston, SC 29406, USA; zchen88@gmail.com (Z.C.); bgreene236@gmail.com (E.D.G.); lcampbell@t3-tissuetestingtechnologies.com (L.H.C.); 2 Department of Bioengineering, Clemson University, Charleston, SC 29425, USA; 3 Department of Regenerative Medicine and Cell Biology, Medical University of South Carolina, Charleston, SC 29425, USA; 4 Department of Mechanical Engineering, University of Minnesota, Minneapolis, MN 55455, USA; bischof@umn.edu (J.C.B.); gaoxx656@umn.edu (Z.G.)

**Keywords:** heart valve, cryopreservation, vitrification, nanowarming, complex tissue preservation

## Abstract

Heart valve cryopreservation was employed as a model for the development of complex tissue preservation methods based upon vitrification and nanowarming. Porcine heart valves were loaded with cryoprotectant formulations step wise and vitrified in 1–30 mL cryoprotectant formulations ± Fe nanoparticles ± 0.6 M disaccharides, cooled to −100 °C, and stored at −135 °C. Nanowarming was performed in a single ~100 s step by inductive heating within a magnetic field. Controls consisted of fresh and convection-warmed vitrified heart valves without nanoparticles. After washing, cell viability was assessed by metabolic assay. The nanowarmed leaflets were well preserved, with a viability similar to untreated fresh leaflets over several days post warming. The convection-warmed leaflet viability was not significantly different than that of the nanowarmed leaflets immediately after rewarming; however, a significantly higher nanowarmed leaflet viability (*p* < 0.05) was observed over time in vitro. In contrast, the associated artery and fibrous cardiac muscle were at best 75% viable, and viability decreased over time in vitro. Supplementation of lower concentration cryoprotectant formulations with disaccharides promoted viability. Thicker tissues benefited from longer-duration cryoprotectant loading steps. The best outcomes included a post-warming incubation step with α-tocopherol and an apoptosis inhibitor, Q-VD-OPH. This work demonstrates progress in the control of ice formation and cytotoxicity hurdles for the preservation of complex tissues.

## 1. Introduction

Preservation constraints place enormous logistical burdens on transplantation, regenerative medicine, research, and other areas spanning medicine. Methods for the long-term cryopreservation or “banking” of viable tissues would revolutionize transplantation [1,2]. Unfortunately, the banking of complex living tissues using current tissue banking practices employing conventional cryopreservation by freezing is not feasible. This is due to the damage caused by intra and extra-cellular ice formation that leads to cell death [3]. Refrigerated storage for most organ types is impacted by ischemia within 8–12 h or sooner. An alternative cryopreservation approach is known as vitrification [4,5,6,7,8,9,10]. Vitrification—cryopreserved storage in a “glassy” rather than crystalline phase—is an important approach for enabling tissue banking and regenerative medicine, offering the ability to store and transport cells and tissues for biomedical uses [6,11,12]. In ice-free cryopreservation by vitrification, the formation of ice is prevented by the presence of high concentrations of chemicals known as cryoprotectants (CPAs) that both interact with and replace water and, thereby, minimize the opportunity for water molecules to nucleate to form ice during cooling. Ice-free vitrification below the glass transition temperature (Tg), −120 °C, has been used successfully to maintain the viability and function of small-scale tissue samples, such as human oocytes where it has revolutionized clinical in vitro fertilization practice [13], short lengths of blood vessels [12], tumor samples [14], and more recently hepatocytes in droplets [15]. The survival of a single vital mammalian organ after vitrification and transplantation was reported by Fahy and colleagues in 2009 [16]. However, the reproducible practical application of vitrification has been limited to cell suspensions, droplets, and thin tissues in volumes < 1–3 mL due to diffusive (heat and mass transfer) and phase change limitations that have historically, with the exception of a single rabbit kidney [16], precluded the use of vitrification for preservation of larger tissues and organs [9,17,18].

To circumvent this problem, thereby permitting the vitrification of larger sample volumes, there have been two recent discoveries. First, nanowarming—the radiofrequency (RF) excitation of magnetic nanoparticles (mNPs) in a cryoprotectant solution—was developed by the Bischof group at the University of Minnesota to aid in the volumetric rewarming of tissues [19,20,21]. This approach relies on uniform heat generation in close proximity to the biomaterial and overcomes the fundamental limitations experienced with convective boundary or microwave warming [18,21]. This innovative rewarming technique increases warming rates by an order of magnitude or more over conventional boundary warming, minimizing the risks of ice nucleation during rewarming, and does not depend on the size of the sample [21]. Therefore, obstacles to scaling up are significantly reduced using nanowarming [20,21,22]. 

The second discovery was the impact of disaccharide supplementation of the 55% CPA formulation known as VS55 on ice nucleation [22,23,24]. The addition of 0.4–0.6 M sucrose or trehalose to VS55 reduces the risks of ice formation during rewarming [22] without visible signs of ice after 7 days at −80 °C [23]. The ice-free vitrification of porcine pulmonary heart valves with VS55 supplemented with 0.3 M sucrose plus 0.3 M trehalose has been our best formulation for ice-free viable vitrification of heart valves in a total volume of 30 mL [23]. In this tissue model, Fe nanoparticles are distributed around and within the lumen of heart valves to obtain rapid tissue warming from vapor phase nitrogen temperatures. The viability of the tissue components of porcine pulmonary heart valves after rewarming by convection boundary warming or by nanowarming was assessed using the alamarBlue metabolic assay. There was no significant statistical difference between nanowarmed and convection-warmed leaflets. However, the nanowarmed conduit and muscle band at the base of the valve were both significantly higher than convection-warmed valves (*p* < 0.05, *t*-test; [23]). The presence of disaccharides appeared to reduce the dependence on nanowarming for retention of tissue cell viability, but nanowarming was still needed.

This manuscript is focused on studies for the further optimization of these cryopreservation and warming methods, combining disaccharides with ice-free preservation formulations (Table 1) with and without nanowarming to enable viable heart valve preservation and warming. Two hypotheses were tested. First, that the combination of disaccharides and nanowarming will permit an overall reduction in cryoprotectants. Second, that the post-warming treatment of ice-free vitrified heart valve tissues with an antioxidant and an apoptosis inhibitor under physiological conditions will improve cell viability.

## 2. Materials and Methods

### 2.1. Tissue Procurement and Preparation

We used porcine pulmonary and aortic heart valve tissue models. These heart valves are inexpensive complex tissues with three tissue components (leaflets, conduit, and cardiac muscle) that do not require the killing of animals specifically for research studies. We can obtain a number of hearts simultaneously, which permits the study of multiple samples in one experiment. In these experiments, we collected pig hearts from a local food processing plant. The hearts were handled in a similar manner as human hearts are for transplant from that point forward. The hearts were rinsed and placed in ice cold phosphate buffered saline (PBS) and transported on ice. Upon receipt at the lab, the valves were harvested and dissected as they would be if they were to be used for heart valve replacement. One valve from each batch was processed immediately as a “fresh control” and the rest were employed for the experimental groups and rewarmed after a period of storage below −130 °C in a mechanical storage freezer. The control valves and experimental valves, after rewarming, were dissected into 9 pieces of leaflet with associated artery and cardiac muscle. The tissues were then further separated into their individual components for viability assessment.

### 2.2. Cryopreservation Solution Preparation

All solutions used deionized water (prepared with an activated carbon filter, mixed bed working deionizer, and a mixed bed polishing deionizer to attain a sensitivity of 10 MΩ cm at room temperature). The solutions were prepared with raw materials that met or exceeded the requirements of the American Chemical Society (www.ACS.org accessed 1 October 2021). If no such specifications exist, chemicals of the highest purity available were used. The solutions were made at room temperature and stored at 4 °C for up to 4 weeks. The solution formulations described in Table 1 are for 1 L batches manufactured as previously described [8,23].

### 2.3. Cryoprotectant (CPA) Formulations Used for Vitrification

Table 1 provides the CPA formulations we employed as vitrification solutions, which we refer to as VS. The original baseline formulation was VS55 with 8.4 M, 55% CPAs. VS55 minimizes ice formation provided that the cooling and warming rates are as fast as possible using convection warming for 1–3 mL samples. VS83 is a higher concentration of the same CPAs; however, it was not used in these studies because it is too toxic for cell survival for viable tissue applications [25]. The exception is articular cartilage where tissue permeability restricts CPA access and good chondrocyte survival strategies have been developed employing VS83 [26]. We are currently investigating applications of VS83 where extracellular matrix preservation is required without cell viability (examples included skin, tendon, and decellularized tissues). VS49 is a lower concentration, 49%, of the same CPAs. At 49% CPAs, the CPA concentration is below the 55% required to prevent ice formation during the cooling and warming of samples. We routinely use VS49 to screen for new compounds that promote ice-free vitrification. DP6 (3 M DMSO plus 3 M propylene glycol, 6 M) avoids formamide use, a compound that may be causing some loss of viability in our VS55-based protocols. This solution is even further below the 55% concentration range required to minimize ice formation for samples in the 1–3 mL range. Disaccharides, sucrose or trehalose, were used to supplement these solutions (Table 1). High concentrations of each disaccharide (0.6 M) or a combination (equimolar concentrations totaling 0.6 M) were used. Disaccharides, when used, were added in the last CPA addition step. Similarly, Fe nanoparticles, when used, were added in the last CPA addition step.

### 2.4. CPA Addition and Removal

For pulmonary heart valves, sequential VS addition and removal solutions were used. The VS chemical component concentration was increased from ⅛, ¼, ½, ¾ to 100% of the final full strength VS formulation employed. The diluted VS solutions were prepared by the addition of appropriate volumes of 1× Euro–Collins base solution (EC). The solutions employed for removal were similar to those employed for addition except that 200–400 mM mannitol was added to the formulation as an osmotic buffer. For example, using seven steps, the VS CPAs were decreased from full strength to 0 M CPA plus mannitol to 0 M CPA, plain EC, and finally Dulbecco’s modified eagle medium (DMEM). Additional work on aortic heart valves was also carried out via the same step-loading method but with increasing DP6 concentrations and VS55 + 0.3 M sucrose and 0.3 M trehalose in the last loading step.

### 2.5. Silica-Coated Iron Oxide Nanoparticles (Coated mNPs) Synthesis

**Figure 1:**
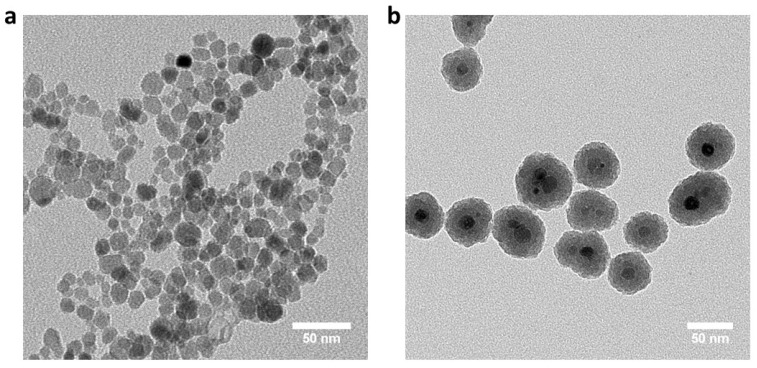
Representative transmission electron microscopy images of nanoparticles (**a**) EMG308 Fe nanoparticles and (**b**) silica-coated EMG308 nanoparticles.

The coated mNPs were synthesized using a previously published procedure [27]. Briefly, 1.440 g Fe EMG308 (Figure 1a) was added to a pre-dissolved polyvinylpyrrolidone (48 g, PVP10, average molecular weight 10,000) aqueous solution (total water volume is 432 mL) and subsequently probe sonicated (Q500, Qsonica. Newtown, CT, USA) for 45 min. Then, the mixture was mixed with 3.2 L ethanol by probe sonication for 45 min while stirring. A total of 160 mL ammonia was added to the mixture while stirring with an overhead mechanical stirrer (OS20-S Waverly, SoCal BioMed, Forest, CA, USA), and 60 mL of tetraethylorthosilicate (Sigma Aldrich, St. Louis, MO, USA) was added while stirring. After 1 h, 15 mL 2-[methoxy(polyethyleneoxy)-propyl]9–12-trimethoxysilane (Gelest, Morrisville, PA, USA) was added to the mixture, and 2.25 mL chlorotrimethylsilane (Sigma Aldrich, St. Louis, MO, USA) was added 30 min later. The reaction continued overnight and the coated mNPs were collected and purified via repeated centrifugation. Each batch of coated mNPs was characterized by transmission electron microscopy (Tecnai T12, FEI, TSS Microscopy, Hillsboro, OR, USA). A representative batch of coated mNPs is shown in Figure 1b.

### 2.6. CPA Exposure Toxicity on Aortic Heart Valves

Conduit, muscle, and leaflets of aortic heart valves were exposed to DP6 and VS55 + 0.3 M sucrose and 0.3 M trehalose via the step-loading and removal procedure described above. The step duration was varied between 15, 30, and 60 min. The viability of the CPA-exposed samples was analyzed by alamarBlue.

### 2.7. Cooling Steps and Storage

Two steps were used for cooling samples. First, rapid cooling to ~−100 °C at 6.8 °C/min was achieved by placing samples in a precooled (−135 °C) 2-methylbutane bath. Then, slow cooling was performed by placing the vials in air in the top of a storage freezer, either a −135 °C mechanical freezer or near the top of a vapor-phase nitrogen-cooled freezer. The samples were then stored at or below −135 °C for at least 1 day before warming. Please see Brockbank et al. [8,23] for more details on ice-free vitrification methods.

### 2.8. Convection Warming

Heart valves cryopreserved without Fe were warmed using a two-stage process consisting of slow warming to −100 °C (5 min) followed by rapid as possible warming to melting (3 min and 40 s). The slow warming rate was created by placing the sample at the top of a −135 °C freezer and the more rapid warming rate was generated by placing the sample in a 40 °C water bath. The tissues were removed from their packaging for stepwise CPA removal when they achieved approximately −25 °C. The convection-warmed samples were controls for comparison with the mNP RF nanowarmed samples. 

### 2.9. Nanowarming

The final CPA concentration during CPA addition, prior to cryopreservation, contained 7.6 or 10 mg/mL Fe nanoparticles (EMG308, Ferrotec, Santa Clara, CA, USA). Pulmonary valve rewarming was performed in a single step by placing the samples in a radio frequency coil and heating with a 6.0 kW terminal, 150–400 kHz, EASYHEAT 5060LI solid state induction power supply with a remote work head and custom multi-turn helical coil with 6–7 turns to create a 35 kA/m magnetic field in the center (Ambrell, Rochester, NY, USA). After ~1 min and 40 s, the pulmonary valves reached approximately −25 °C. The aortic heart valve components were placed in a 1.2 mL cryovial (Thermo Fisher Scientific, Waltham, MA, USA) with 1 mL VS55 containing 10 mg Fe/mL coated mNPs, 0.3 M sucrose, and 0.3 M trehalose. The aortic heart valves were nanowarmed in a 1 kW Hotshot inductive heating system with a 2.75-turn, water-cooled copper coil (Ameritherm Inc., Scottsville, NY, USA) until the sample reached −20 °C. Stepwise CPA removal was then performed as described above. 

### 2.10. Post Warming Treatment

In select experiments, the heart valve tissue components were placed in culture medium with a combination of supplements, 25 µM Q-VD-OPH (QVD, MP Biomedical, Irvine, CA, USA) and 100 µM α-tocopherol group (vitamin E, Sigma Aldrich, St. Louis, MO, USA), during physiological tissue culture for 4 h before testing with alamarBlue.

### 2.11. Viability Assessment

An alamarBlue™ resazurin assay was used to evaluate the metabolic activity of control and treated tissue samples [21,28]. After stepwise CPA removal, the valves were rinsed twice with DMEM + 1% pen-strep and then dissected in preparation for viability testing. Tissue samples were incubated in 2 ml of DMEM + 10% FBS culture medium for one hour to equilibrate, followed by the addition of 20% alamarBlue under standard cell culture conditions for 3 h. A 4 h preincubation was used for experiments in which post-warming supplements were evaluated. AlamarBlue is a non-toxic fluorometric indicator based on the detection of metabolic activity. Fluorescence was measured at an excitation wavelength of 544 nm and an emission wavelength of 590 nm. In some experiments, this evaluation was performed after 24 and 48 h to further characterize the behavior of the cells in the re-warmed tissues. After each reading, the tissues were washed with PBS and placed back into tissue culture. The results shortly after rewarming (day 0) demonstrate cell viability. Decreases over 1–2 days of culture are indicative of cell death due to apoptosis and increases are due to cell recovery or proliferation. The control and experimental tissues were dried at the end of each experiment and the results expressed as relative fluorescence units (RFU)/mg tissue dry weight.

### 2.12. Statistical Methods

All experiments were performed several times. Statistical analysis was performed by one-way ANOVA with Tukey’s multiple comparison tests or by *t*-test as appropriate for the type of data being analyzed.

## 3. Results and Discussion

Historically, the major limitations of vitrification for large tissue samples have been potential cytotoxicity due to prolonged exposure to the cryoprotectants employed and ice-formation during rewarming. We emphasize rewarming over cooling because the risks of ice nucleation and growth are an order of magnitude greater during warming than cooling [21]. Employing CPA concentrations above 55% is difficult due to CPA cytotoxicity. However, the use of the lower concentration CPA formulations used for small samples, such as oocytes, is not feasible because the critical cooling rate for avoiding ice nucleation during cooling cannot be achieved. In tissue models, we have resorted to adding the highest CPA concentrations during step-wise CPA addition and initiating the dilution of the CPAs during rewarming at sub-zero temperatures. Rewarming is usually performed at room temperature in a bath. Once the tissue has reached approximately −20 °C, it is moved to an ice bath and CPA removal by washing is initiated. Rewarming in a 37 °C water bath works well for small samples (≤3 mL), but larger samples experience ice nucleation due to devitrification during rewarming because conventional boundary warming is not fast enough—in the range of Td. It is necessary that heat generation is uniform. The most well-recognized attempts at achieving uniform heat generation employed microwave rewarming [29,30,31,32,33,34,35,36,37]. Unfortunately, variability in the resonance of the cavity and the geometric variability of dielectric properties within tissue samples have not yet led to uniform rapid rewarming, likely due to “hot spots” within the samples. Thus, microwave technology has consistently failed to adequately warm tissues. The discovery of nanowarming by the Bischof group and its application to cardiovascular tissue models [21] opened the opportunity to vitrify larger samples. In the same timeframe, we discovered the benefits of supplementing VS55 with sucrose and/or trehalose [22,23]. While rapid cooling and rewarming rates promote vitrification, the resulting non-uniform cooling and rewarming across the specimen can give rise to thermomechanical stresses that may cause structural damage. Increasing the CPA solution concentration of VS55 with ≥0.4 M of these sugars suppresses the critical cooling and rewarming rates for avoidance of ice nucleation (ice does not form), potentially decreasing the thermomechanical stress effects. Publication was delayed due to the need to file patents [38,39]. A major objective in the experiments presented here was to determine whether or not nanowarming was still needed as further process optimization was performed.

### 3.1. The First Group of Experiments Evaluated the Hypothesis That a Combination of Disaccharides and Nanowarming Will Permit an Overall Reduction in Cryoprotectants

**Figure 2:**
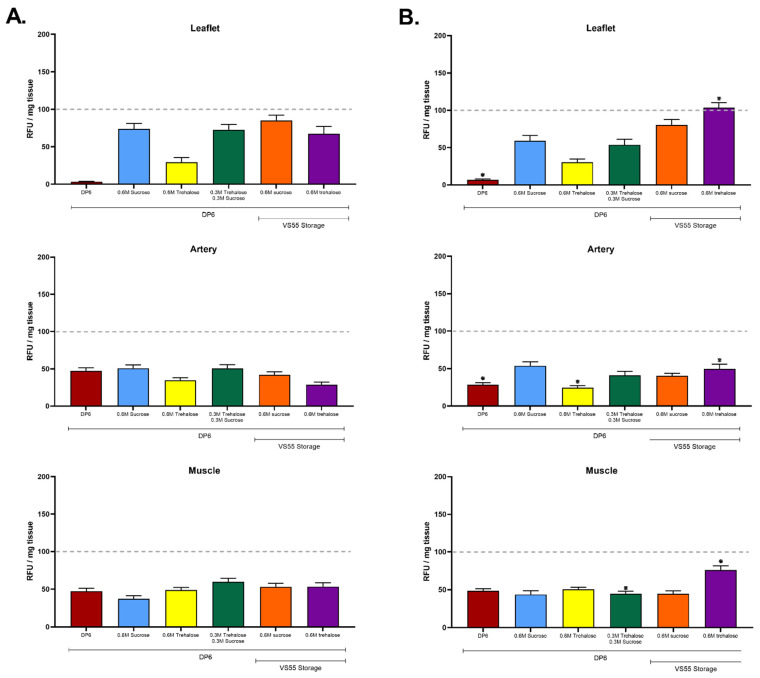
Ice-free vitrification of pulmonary heart valves comparing impact of loading with DP6 on viability after vitrification in DP6 + disaccharides or VS55 + disaccharides. (**A**) No Fe, with boundary warming. (**B**) Nanowarmed with Fe. The top graph in each case is leaflets, center is pulmonary artery, and the bottom is fibrous muscle band at the base of the heart valve. Viability was assessed using alamarBlue. The best outcomes were observed with VS55 + 0.6 M trehalose and nanowarming for each tissue type. The results are shown as the mean ± 1 standard error, *p* < 0.05. * comparison of ±Fe.

The experiments shown in Figure 2 also evaluated whether or not (i) loading strategies that avoided or minimized exposure to formamide might have a benefit and (ii) whether or not disaccharides might make the final solution more effective for the preservation of cell viability. Sucrose and trehalose were employed because they have been shown to stabilize the glassy state, minimizing the risks of devitrification, ice nucleation, and ice-induced damage during rewarming [22,23]. The results demonstrate the feasibility of loading with DP6 prior to vitrification in either DP6 with sugars or VS55 formulations with sugars (Figure 2A,B). The presence of sugars in DP6 had a clear benefit for the preservation of leaflet viability (*p* > 0.05) but not conduit or cardiac muscle. Two DP6 supplemented convection-warmed groups were not significantly different compared with VS55 supplemented convection-warmed groups (Figure 2A). The overall best outcome was a nanowarmed group in which the DP6 loading was followed by cryopreservation in VS55 + 0.6 M trehalose (Figure 2B). This group also had the highest cardiac muscle viability (75.93%) and will be further evaluated in future research focused on heart cryopreservation. There was no significant difference for convection versus nanowarming using 0.6 M sucrose. These results support the hypothesis that a combination of disaccharides may permit a 2.4 M reduction in CPA concentration and that nanowarming has a slight advantage over convection warming using 30 mL volumes in 3 cm diameter tubes (Figure 2). Based upon modeling [22], we expect even larger volume and/or diameter tubes will lead to lower convective rates while maintaining nanowarming rates, thereby showing an even greater advantage for nanowarming upon scaling up.

**Figure 3:**
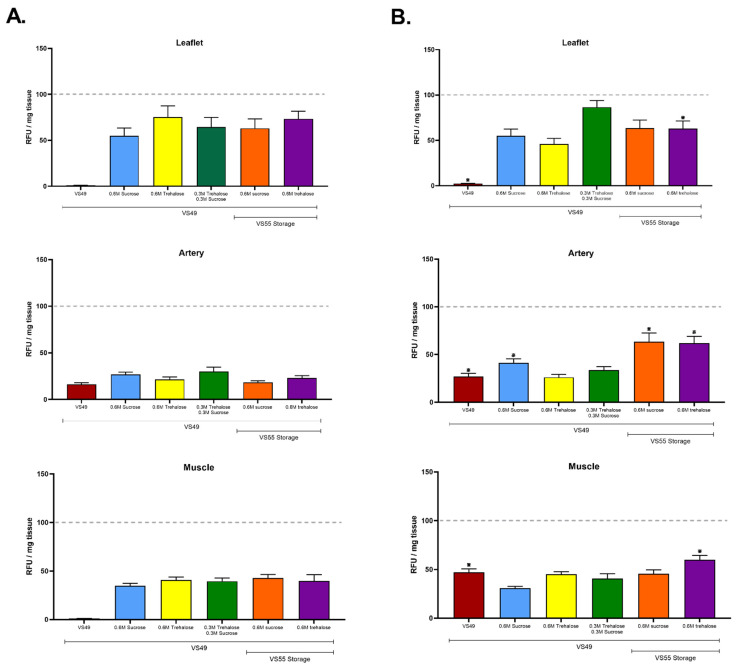
Ice-free vitrification of pulmonary heart valves comparing the impact of loading with VS49 on viability after vitrification in VS49 + disaccharides or VS55 + disaccharides. (**A**) No Fe, with boundary warming. (**B**) Nanowarmed with Fe. The top graph in each case is the leaflet, the center is the pulmonary artery, and the bottom is the fibrous muscle band at the base of the heart valve. Viability was assessed using alamarBlue. The best viability outcomes were observed employing nanowarming for each tissue type. The results are shown as the mean ± 1 standard error, *p* < 0.05. * comparison of ±Fe.

Figure 3 summarizes similar experiments with VS49 in which formamide was present. The results demonstrate that neither VS49 (Figure 3) nor DP6 (Figure 2) support cell viability unless supplemented with disaccharides. What was clear in both sets of experiments was that the addition of sucrose, trehalose, or a combination of the two sugars had a significant benefit compared with leaflets vitrified with either DP6 or VS49 alone. In marked contrast, minimal differences were observed for the arterial conduit or fibrous cardiac muscle tissue samples. The highest mean value for VS49 nanowarmed leaflets was with the combination of sucrose and trehalose (*p* < 0.05), but there was no significant difference compared with convection warming. The highest mean value for VS49 convection-warmed leaflets was with trehalose, significantly higher than the equivalent nanowarmed group (*p* < 0.05), but there were no significant differences compared with the other convection-warmed sugar supplemented groups. These experiments support the hypothesis that a combination of disaccharides may permit a 0.9 M reduction in CPA concentration. Again, nanowarming had a slight, but not significant in this case, advantage over convection warming in these experiments performed using 30 mL volumes in 3 cm diameter tubes. The range of tissue viabilities was the same in the two sets of experiments, suggesting that the removal of formamide had little or no impact on final cell viability. However, since the highest mean leaflet values comparing the DP6 and VS49 experiments were observed with the DP6 loading and VS55 plus sucrose for convection warming (80.39%) and plus trehalose for nanowarming (103.44%), these results led to the prioritization of a DP6 stepwise approach for CPA addition for further studies in combination with VS55 plus disaccharides in the final CPA addition step. 

### 3.2. The Second Series of Experiments Examined the Hypothesis That Post-Warming Treatment of Ice-Free Vitrified Heart Valve Tissues Will Improve Cell Viability

**Figure 4:**
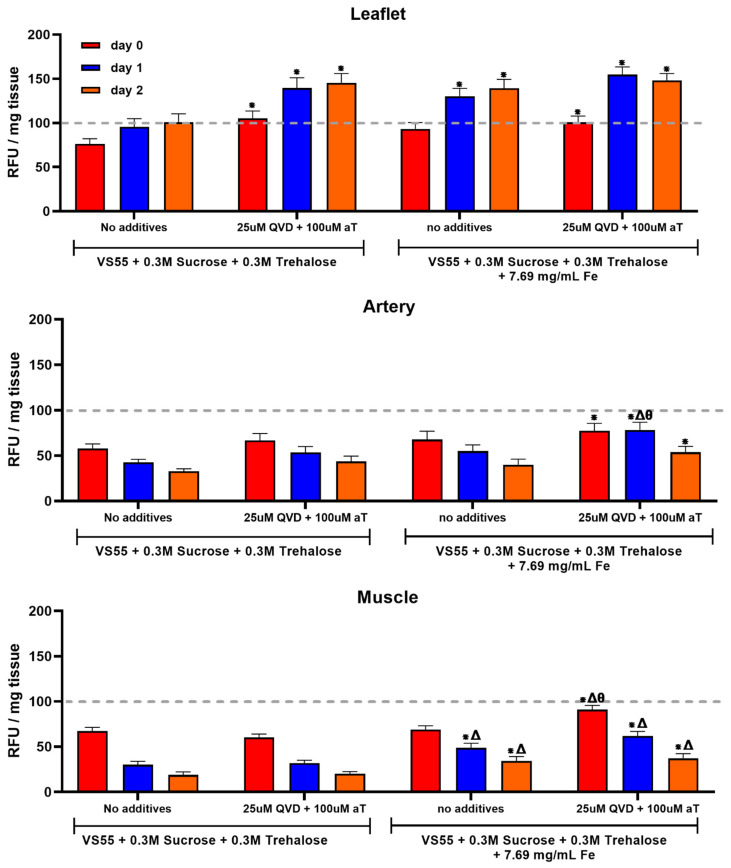
Impact of post-rewarming treatment with an apoptosis inhibitor and antioxidant upon tissue viability. Pulmonary valves were vitrified in VS55 + 0.3M sucrose and 0.3M trehalose and warmed using either conventional boundary warming (**left**) or nanowarming (**right**). The top graph in each case is the leaflet, the center is the pulmonary artery, and the bottom is the fibrous muscle band at the base of the heart valve. The heart valve tissue components were then cultured under physiological conditions with and without supplements, 25 µM QVD and 100 µM α-tocopherol, for 4 h, 24 h, and 48 h with viability assessment at each time point using alamarBlue. The results are shown as the mean ± 1 standard error, *p* < 0.05 * compared to no Fe or supplements, Δ compared to supplements only, and θ compared with no supplements + Fe.

Heart valves were warmed using either nanowarming or convention warming. CPA removal was conducted using the DP6 stepwise method using 10 min incubation steps of decreasing CPA concentration. The heart valve tissue components were then placed in culture medium with a combination of supplements, 25 µM Q-VD-OPH (QVD) and 100 µM α-tocopherol group (vitamin E), during physiological tissue culture for 4 h and then tested using alamarBlue. The metabolic activity was assessed again on days one and two. After the first and second alamarBlue readings, the tissues were rinsed and placed back in media with the supplements. The nanowarmed valves demonstrated a higher metabolism (viability) in the conduit and muscle samples than in conventionally convection-warmed samples. There were no significant differences between leaflets from the conventionally warmed and nanowarmed valves when the post-rewarming supplements were employed (Figure 4). Nanowarming combined with supplements post-warming was significantly better than nanowarming without supplements and convection warming with or without supplements for both conduit and muscle. The leaflets remained viable over the two days of in vitro follow up (Figure 4). In contrast, the other tissues demonstrated a progressive loss of cell viability (Figure 4). These results support the hypothesis that post-rewarming supplements have a positive impact on cell survival using the VS55 formulation with disaccharides. Furthermore, at the volume employed (30 mL), these results also suggest that there is a need for nanowarming for the thicker tissue components, artery, and the fibrous cardiac muscle band where the CPA concentration achieved is likely less than in the relatively thin leaflets. The leaflets were well preserved with a viability similar to untreated fresh leaflets over the days post-warming in vitro. Convection-warmed leaflet viability was not significantly different from that of nanowarmed leaflets without supplements immediately after rewarming; however, a significantly higher nanowarmed leaflet viability was observed on days one and two in vitro. In contrast, the pulmonary artery and fibrous cardiac muscle were at best 75% viable, and the cell viability decreased on days one and/or two in vitro. This observation could be a function of suboptimal culture methods, but we believe it is most likely due to the different preservation method requirements for the three tissue types in heart valves. Further studies are being focused on improving the ice-free preservation of thicker-walled arteries, such as the pulmonary artery and aorta, and cardiac muscle.

**Figure 5:**
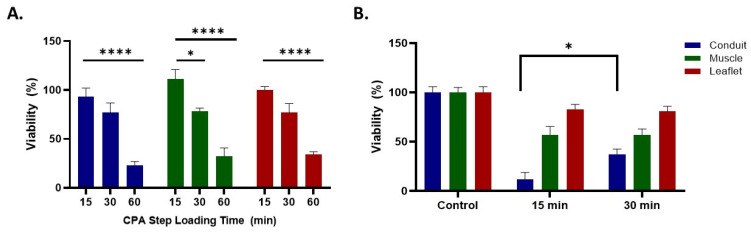
Aortic valve viability after loading, CPA exposure, and rewarming. (**A**) CPA exposure toxicity on heart valve tissues. Heart valve was exposed to CPA in stepwise manner for various time intervals. Longer exposure reduced the viability of the samples due to CPA toxicity. N = 5–11 (**B**) Heart valve nanowarming recovery viability using different CPA loading durations. N = 6–15. Viability was assessed using alamarBlue. Statistical significance is indicated with asterisks: * *p* < 0.05; *p* < 0.001; **** *p* < 0.0001. The one-way and two-way analysis of variance (ANOVA) with Tukey’s multiple comparison tests (GraphPad Prism, GraphPad^®^ Software, Inc., San Diego, CA, USA) were performed on data presented as the mean ± 1 standard error.

Finally, aortic heart valves were studied. Aortic conduits are thicker compared to pulmonary ones, and therefore are more challenging to cryopreserve due to the lower cryoprotectant concentration diffusivity to the center of the tissue [40]. To increase the cryoprotectant concentration in the thicker tissue, one solution is to increase the loading time. However, cryoprotectants are highly toxic [41] and extending exposure to cryoprotectants will cause a significant reduction in tissue viability. Our previous loading step duration was 15 min, employing porcine carotid artery (~1 mm in thickness) [21]. Figure 5A shows the viability of aortic heart valve components after increasing the loading step duration. As shown in Figure 5A, the 15 and 30 min loading step procedures did not cause a significant reduction in viability in either the conduit or leaflet but the 30 min loading step slightly reduced muscle viability. However, the 60 min loading step procedure significantly compromised the viability all three aortic heart valve tissues. Therefore, the 60 min loading procedure was abandoned due to the high toxicity effect. We nanowarmed aortic heart valves after both the 15 min loading step and 30 min loading step methods with coated mNPs, because coated MNPs previously showed a better heating performance in cryoprotectants compared to EMG308 [27]. Figure 5B shows the aortic heart valve component viability after vitrification and nanowarming. The viability of muscle and leaflet were the same in both procedures, yet a significant increase in the viability of the conduit was observed after the longer cryoprotectant loading duration. This is most likely due to the higher cryoprotectant concentration in the conduit after a 30 min loading step, and therefore the reduced possibility of ice nucleation during rewarming compared to the 15 min loading step method. In summary, increasing the cryoprotectant loading duration increased thick tissue—aorta—viability after vitrification and rewarming. We would expect an even higher viability when we increase the heating rate by either increasing the RF frequency or field strength, or using a higher coated mNP concentration.

It is likely that ice-free vitrification may benefit from new designer low molecular weight cryoprotectants, such as those being developed by the Ben group at the University of Ottawa [42], or in combination with other new preservation technologies in development [43]. For example, it may eventually be possible to harness the engineering innovations for the perfusion and supercooling of livers recently demonstrated by the Uygun group at Massachusetts General Hospital [44,45] to take organs to the ultralow cryogenic temperatures required for vitrification. Such an achievement would inaugurate a completely different paradigm for organ transplantation [46], including complex vascularized tissues such as limb extremities.

This work demonstrates progress towards the control of ice formation and cytotoxicity hurdles for ice-free cryopreservation of a large complex tissue model by the application of nanowarming, disaccharides, and post-warming supplements that minimize oxidation and apoptosis. Further investigation of alternative antioxidants and apoptosis inhibitors may lead to even better outcomes. Nanowarming is needed for optimal heart valve leaflet preservation and is even more important for the other heart valve tissue components. These advances will lead to the translation and banking of living human complex allotransplants. The establishment of such banks will go far beyond simply providing on demand tissues and organs with better genotypic and phenotypic matching (size, age, color, gender). The availability of banked allografts will also result in better immunological outcomes by optimizing donor and recipient human leukocyte antigen matching and minimizing the risks of graft versus host disease. The long-term storage of healthy tissue is a foundational technology that could become a cornerstone of biomedicine. For example, the simultaneous banking of bone marrow from donors will permit existing advances in tolerance induction in animal models to be translated to humans, further minimizing the need for immune system regulation post-transplant. The potential benefits of long-term storage using ice-free cryopreservation are wide spread and diffuse, enabling advances in many areas of public health spanning cancer treatment, organ transplantation, trauma care, disaster preparedness, tissue engineering, drug screening and disease modeling, regenerative medicine, and more [1,2]. 

## 4. Conclusions

These studies demonstrate that nanowarming is still superior to convection warming after the addition of disaccharides to VS55, and that the addition of disaccharides also permits a reduction in cryoprotectant concentrations with the retention of cell viability. Other protocol steps that improve tissue viability include longer cryoprotectant loading incubation steps for thick tissue viability and post-warming incubation with vitamin E, an antioxidant, and QVD, an apoptosis inhibitor.

## Figures and Tables

**Table 1:** CPA Formulations.

	VS49	VS55	DP6	Units
Component	Quantities	Quantities	Quantities	
5X EuroCollins	200	200	200	mL
HEPES buffer	2.39	2.39	2.39	Grams
Propylene Glycol *	150.011	168.38	228.27	Grams
Formamide *	124.34	139.56	0	Grams
DMSO *	215.72	242.14	234.39	Grams
Water	To 1 L	To 1 L	To 1 L	mL
pH	7.9–8.1	7.9–8.1	7.9–8.1	Units
Osmolality **	400 ± 5	460 ± 5	325 ± 5	mOsm
CPA Moles	7.48 M	8.4 M	6 M	

* Liquids measured by weight. ** 20× dilution = 50 µL in 950 µL of distilled water.
